# Lenalidomide and pomalidomide modulate hematopoietic cell expansion and differentiation in the presence of MSC

**DOI:** 10.1007/s12185-024-03815-y

**Published:** 2024-07-12

**Authors:** Sumie Fujii, Yasuo Miura

**Affiliations:** 1 Department of Transfusion Medicine and Cell Therapy, Kyoto University Hospital, Kyoto, 606-8507 Japan; 2 Department of Transfusion Medicine and Cell Therapy, Fujita Health University School of Medicine, Toyoake, Aichi 470-1192, Japan

**Keywords:** Mesenchymal stem cell, Lenalidomide, Cytopenia

## Abstract

Cytopenia is a well-documented complication in the treatment of hematological malignancies with lenalidomide and pomalidomide. Although prior studies have highlighted direct effects on hematopoietic cells to explain this adverse effect, the involvement of hematopoietic-supportive stroma remains less understood. This study examined the effects of lenalidomide/pomalidomide on the expansion and differentiation of human CD34^+^ hematopoietic stem/progenitor cells (HSPCs) in vitro, in co-culture with human bone-marrow mesenchymal stromal/stem cells (MSCs). Our findings indicate that lenalidomide/pomalidomide increases the population of immature CD34^+^CD38^−^ cells while decreasing the number of mature CD34^+^CD38^+^ cells, suggesting a mechanism that inhibits early HSPC maturation. This effect persisted across myeloid, megakaryocytic, and erythroid lineages, with MSCs playing a key role in preserving immature progenitors and inhibiting their differentiation. Furthermore, in myeloid differentiation assays augmented by granulocyte-colony stimulating factor, lenalidomide/pomalidomide not only enhanced the presence of CD34^+^ cells with mature myeloid markers such as CD11b but also reduced the populations lacking CD34 yet positive for these markers, irrespective of MSC presence. Thus, while MSCs support the presence of these immature cell populations, they simultaneously inhibit their maturation. This finding provides novel mechanistic insights into lenalidomide- and pomalidomide-induced cytopenia, and could guide therapeutic strategies for its mitigation.

**Supplementary Information:**

The online version contains supplementary material available at 10.1007/s12185-024-03815-y.

## Introduction

In the realm of hematologic therapies, lenalidomide (LENA) and pomalidomide (POMA) have emerged as fundamental agents, particularly in the management of multiple myeloma [1, 2] and selected cases of myelodysplastic syndromes [3]. Nonetheless, the administration of these medications is frequently linked with cytopenia, which presents considerable challenges in clinical management. Initial reports from clinical studies using LENA in patients newly diagnosed with myeloma identified incidences of anemia, neutropenia, and thrombocytopenia in approximately 40%, 30%, and 20% of cases, respectively [4]. Similarly, early investigations of POMA in patients with refractory or relapsed myeloma noted comparable adverse effects, with anemia, neutropenia, and thrombocytopenia occurring in about 50%, 40%, and 30% of cases, respectively [2].

Not extensive but noteworthy previous research has explored the cytopenia associated with LENA and POMA. These agents have been shown to impair erythropoiesis by delaying erythroid maturation and promoting the proliferation of immature erythroid cells [5]. Furthermore, they are known to downregulate PU.1, which leads to myeloid maturation arrest and neutropenia [6]. In addition, LENA and POMA have been observed to support the self-renewal of hematopoietic progenitors and increase the proliferation of early megakaryocytic progenitors. However, they concurrently inhibit megakaryocyte maturation and induce thrombocytopenia by degrading IKZF1 and downregulating GATA1 [7]. These findings provide insights into the direct impacts of LENA and POMA on hematopoietic cells, outlining mechanisms that contribute to the observed cytopenia. Nevertheless, the role of hematopoiesis-supporting stroma in these processes remains to be defined.

Hematopoietic cell production within the bone marrow is profoundly influenced by the bone-marrow microenvironment [8], in which mesenchymal stromal/stem cells (MSCs) play a pivotal role [9]. The complex interactions between the hematopoietic system and MSCs, as well as MSC-like cells such as osteo-adipoprogenitors, are essential for effective hematopoiesis [10, 11]. These interactions promote the production of progenitor cells and the processes of myelopoiesis, lymphopoiesis, and megakaryopoiesis, facilitated by both the secretion of soluble factors and the expression of adhesion molecules. This study aims to explore the role of MSCs in the generation of hematopoietic cells from CD34^+^ hematopoietic stem and progenitor cells in the presence of LENA or POMA.

## Materials and methods

### MSC isolation, expansion, and differentiation assays

Human bone-marrow specimens, totalling four, were obtained from AllCells (Alameda, CA). MSCs were isolated from these specimens by first preparing a mononuclear cell suspension at a concentration of 1 × 10^6^ cells/mL [12]. These cells were seeded onto 15 cm culture dishes, and only the adherent cells were further cultured. The culture medium used was Advanced Minimal Essential Medium (Invitrogen/Thermo Fisher Scientific, Waltham, MA), supplemented with 5% fetal bovine serum (FBS, Gibco/Thermo Fisher Scientific), 100 µM ascorbic acid (Wako Pure Chemical Industries, Osaka, Japan), 2 mM L-glutamine, and an antibiotic mixture (100 U/mL penicillin and 100 µg/mL streptomycin, all from Gibco/Thermo Fisher Scientific). After the initial seeding, primary cultures were passaged to promote the expansion of colony-forming units. Cells at passage 3 were used as bone marrow-derived MSCs in this study. For the co-culture experiment, 4.2 × 10^4^ cells were seeded in a 24-well plate. To confirm their MSC identity, surface marker expression was assessed according to the minimal criteria established by the International Society for Cellular Therapy [13]. An overview of the cell isolation process from bone marrow to application is depicted in Figure S1.

For the cell expansion assays, MSCs were cultured at a density of 1.0 × 10^3^ cells per well in 96-well plates and treated with various concentrations of LENA (0, 1, 3, or 10 µM) or POMA (0, 0.3, 1, or 3 µM), both sourced from Chem Scene (Monmouth Junction, NJ). Cell expansion was quantitatively evaluated using the Cell Counting Kit-8 (DOJINDO, Kumamoto, Japan), following the manufacturer's instructions. The growth of cells on days 3, 6, and 9 post-treatment was measured by determining the absorbance at 450 nm, comparing treated cells to untreated controls using a microplate reader. Multilineage differentiation potential of MSCs was evaluated using established assays as described in prior studies [14]. Osteogenic activity was determined by Alizarin Red S staining to evaluate mineral accumulation, based on previously documented methods [15].

### In vivo bone formation assay

MSCs, either untreated or treated with LENA or POMA, were seeded at a density of 1 × 10^6^ cells onto hydroxyapatite/poly (D, L-lactic–co-glycolic acid) scaffolds. These scaffolds, composed of 50 wt% hydroxyapatite and measuring 5 mm in diameter, were supplied by GC Corporation (Tokyo, Japan). According to a standardized protocol, the MSC-loaded scaffolds were incubated at 37 °C in a 5% CO_2_ atmosphere for 1 h before being implanted subcutaneously into non-obese diabetic/severe combined immunodeficiency (NOD/SCID) mice [16]. Ten weeks after implantation, the scaffolds were retrieved, processed for histological examination, and the extent of bone formation was evaluated using hematoxylin and eosin staining. All procedures involving animals were conducted in compliance with the guidelines approved by the relevant institutional review board.

### CD34^+^ cell culture

Human CD34^+^ cells were isolated from bone-marrow mononuclear cells using anti-CD34 immunomagnetic microbeads, and the purity of the CD34^+^ cell population was confirmed via flow cytometry [17]. These cells were cultured in StemSpan Serum-Free Expansion Medium (SFEM) supplemented with 100 ng/mL stem cell factor, 100 ng/mL Fms-related tyrosine kinase 3 ligand, 50 ng/mL thrombopoietin, and 20 ng/mL interleukin-3 at a density of 1.5 × 10^3^ cells per well in a 24-well plate. The culture was conducted in the presence or absence of LENA or POMA. After 10 days of culture, the CD34^+^ cells were analyzed using flow cytometry to assess the composition of the hematopoietic cell populations.

### Myeloid and erythroid induction of CD34^+^ cells

For the induction of myeloid lineages, CD34^+^ cells were initially cultured in SFEM supplemented with the previously mentioned cocktail of cytokines. Subsequently, the culture medium was enriched with granulocyte-colony stimulating factor (G-CSF, 10 ng/mL), and the cells were incubated for an additional period of 7 days. For the induction of the erythroid lineage, CD34^+^ cells were cultured in Iscove's Modified Dulbecco's Medium, supplemented with iron sources (FeSO_4_, 900 ng/mL; Fe(NO_3_)_2_, 90 ng/mL) and an enhanced cocktail of stem cell factor (200 ng/mL), erythropoietin (8000 U/mL), transferrin (12 µg/mL), interleukin-3 (10 ng/mL), and insulin at a density of 9 × 10^3^ cells per well in a 24-well plate [18]. This culture was maintained for 14 days to facilitate erythroid lineage commitment. In co-culture experiments, MSCs were prepared 1 day prior by seeding onto 24-well plates. To examine the effects of LENA and POMA on hematopoietic differentiation, these compounds were introduced into the respective cultures at the start of the induction period.

### Flow cytometric analysis

Flow cytometric analysis was performed on single-cell suspensions derived from either MSC cultures or hematopoietic cell cultures. Cells were stained with fluorescently-conjugated antibodies, as specified in Supplementary Table 1, and subsequently analyzed using a FACS Canto II system. Data were processed using FlowJo software. To ensure accuracy in the analysis, dead cells were excluded from the datasets through staining with propidium iodide, which identifies and removes non-viable cells from the results.

### Quantitative real-time PCR

Quantitative real-time PCR was conducted following established protocols [19]. Total RNA was extracted using the QIAamp RNA Blood Mini Kit (Qiagen, Tokyo, Japan) and subsequently converted to complementary DNA (cDNA) through a reverse transcription process. Each PCR reaction was set up in a 20 µL mixture containing TaqMan Master Mix (Roche, Basel, Switzerland), the prepared cDNA, specific primer pairs, and a TaqMan probe from the Universal Probe Library. Amplification was performed using the StepOne Real-Time PCR System (Applied Biosystems, Waltham, MA). The cycling conditions included an initial denaturation at 95°C for 10 min, followed by 40 cycles of denaturation at 95°C for 15 s and annealing/extension at 60°C for 1 min. Glyceraldehyde-3-phosphate dehydrogenase was used as the internal control to normalize variations in sample loading. The specific primer sets and probes used in this study are detailed in Supplementary Table 2.

### Statistical analysis

Statistical analyses were conducted primarily using Fisher’s exact test, unless otherwise specified. Results are presented as mean ± standard deviation (SD), with statistical significance indicated as follows: *p < 0.05; **p < 0.01.

## Results

### Enhancement of MSC expansion and hematopoietic cell generation from CD34^+^ cells by LENA and POMA

**Fig. 1:**
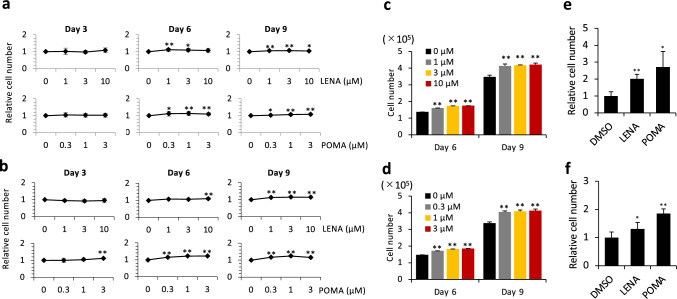
Impact of LENA and POMA on the expansion of MSCs and generation of hematopoietic cells from CD34^+^ cells in vitro. Panels (**a**, **b**) illustrate the expansion profiles over a 9-day period for two distinct batches of human bone marrow mesenchymal stromal/stem cells (MSCs), exposed to increasing concentrations of LENA (0, 1, 3, 10 µM) and POMA (0, 0.3, 1, 3 µM). The expansion was quantitatively assessed using the Cell Counting Kit-8, which measures mitochondrial activity and reflects the number of viable cells, with results displayed relative to DMSO-treated controls. Panels (**c**, **d**) show the expansion of MSCs over a 9-day period, assessed using the trypan blue dye exclusion assay, which directly counts viable cells. Panels (**e**, **f**) detail the number of hematopoietic cells derived from CD34^+^ hematopoietic stem and progenitor cells following a 10-day culture period, treated with 3 µM LENA and 0.3 µM POMA. The enumeration of these cells was quantitatively determined using trypan blue dye exclusion assays, relative to DMSO-treated controls. Statistical significance is indicated by * (*p* < 0.05) and ** (*p* < 0.01)

Our study evaluated the effects of LENA and POMA on the expansion of MSCs and the generation of hematopoietic cells from CD34^+^ cells. In a colorimetric assay using Cell Counting Kit-8, which measures mitochondrial activity and reflects the number of viable cells, treatments with LENA at concentrations of 1, 3, and 10 µM significantly augmented MSC expansion after 9 days of culture (Fig. 1a, b). A similar result was obtained using the trypan blue dye exclusion assay, which directly counts viable cells. In this assay, treatments with LENA at concentrations of 1, 3, and 10 µM also significantly augmented MSC expansion after 9 days of culture (Fig. 1c). Given the absence of apparent differences in expansion across these concentrations, a concentration of 3 µM LENA was selected for subsequent experiments. Similarly, exposure of MSCs to POMA at concentrations of 0.3, 1, and 3 µM resulted in a marked increase in cell proliferation after 9 days (Fig. 1a, b and d). A concentration of 0.3 µM was chosen for further studies due to the lack of considerable variance in expansion among the tested concentrations. The treatment of human CD34^+^ cells with 3 µM LENA or 0.3 µM POMA significantly enhanced the generation of nucleated cells within the StemSpan culture system (Fig. 1e, f). This indicates a potent stimulatory effect of these agents on both MSC expansion and hematopoietic cell proliferation.

### Suppression of MSC differentiation by LENA and POMA

**Fig. 2:**
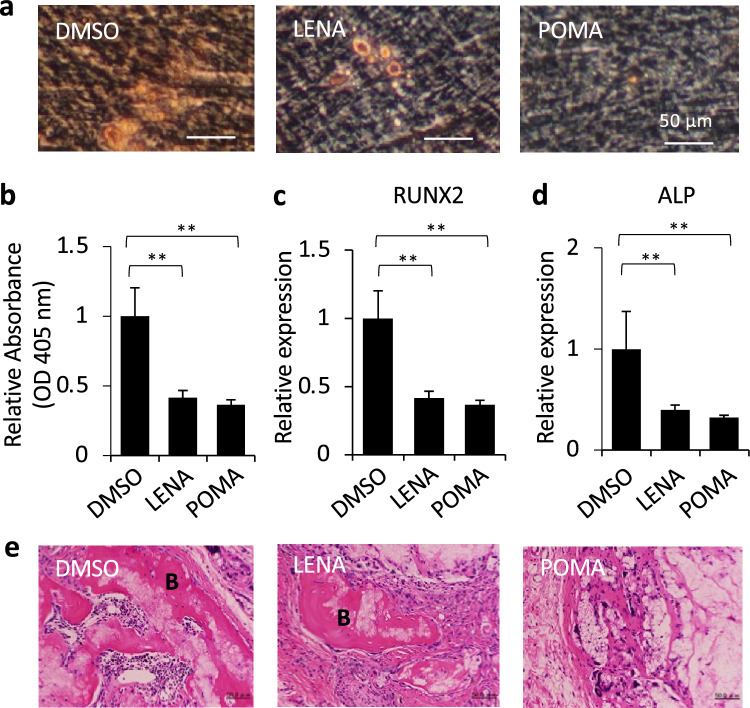
LENA and POMA influence on MSC osteogenic differentiation in vitro and in vivo. **a**, **b** Alizarin Red S staining reveals mineral deposition (visualized as red nodules) in MSCs following a 3-week period of osteogenic induction with treatments of LENA and POMA, compared to DMSO-treated controls (**a**). The extent of mineralization was evaluated quantitatively (**b**). **c**, **d** Quantitative RT-PCR analyses demonstrate the expression levels of osteogenesis-associated markers, runt-related transcription factor 2 (RUNX2) (**c**) and alkaline phosphatase (ALP) (**d**), in MSCs treated with LENA and POMA compared to DMSO controls. **e** In vivo assessment reveals differential bone formation in MSCs treated with LENA or POMA, utilizing a subcutaneous implantation model in immunocompromised mice. Representative images are shown. *n* = 5. Statistical significance is indicated by ** (*p* < 0.01)

Our study explored the impact of LENA and POMA on the differentiation capabilities of MSCs. Following 3 weeks of osteogenic induction in vitro, MSCs subjected to LENA or POMA treatment exhibited reduced mineral deposition, as evaluated by Alizarin Red S staining (Fig. 2a, b). Furthermore, the expression levels of runt-related transcription factor 2 (RUNX2) and alkaline phosphatase (ALP), markers associated with osteogenesis, were found to be lower in treated MSCs compared to control MSCs (Fig. 2c, d). To investigate the bone-forming capability of MSCs in vivo, we utilized a subcutaneous implantation model in immunocompromised mice. Interestingly, MSCs treated with LENA or POMA demonstrated a diminished ability to generate bone compared to control MSCs (Fig. 2e). Regarding adipogenic differentiation, MSCs exposed to LENA or POMA exhibited an increased level of fat deposition, as determined by Oil Red O staining (Fig. S2a–d). Flow cytometric analysis revealed that the surface marker expression pattern of MSCs treated with LENA or POMA remained similar to that of control MSCs (data not shown). In summary, our findings reveal that although LENA- or POMA-treated MSCs meet the phenotypic criteria for mesenchymal stem cells, their differentiation potential is altered by the treatment.

### Enhancement of various hematopoietic cell generation from CD34^+^ cells by LENA and POMA

**Fig. 3:**
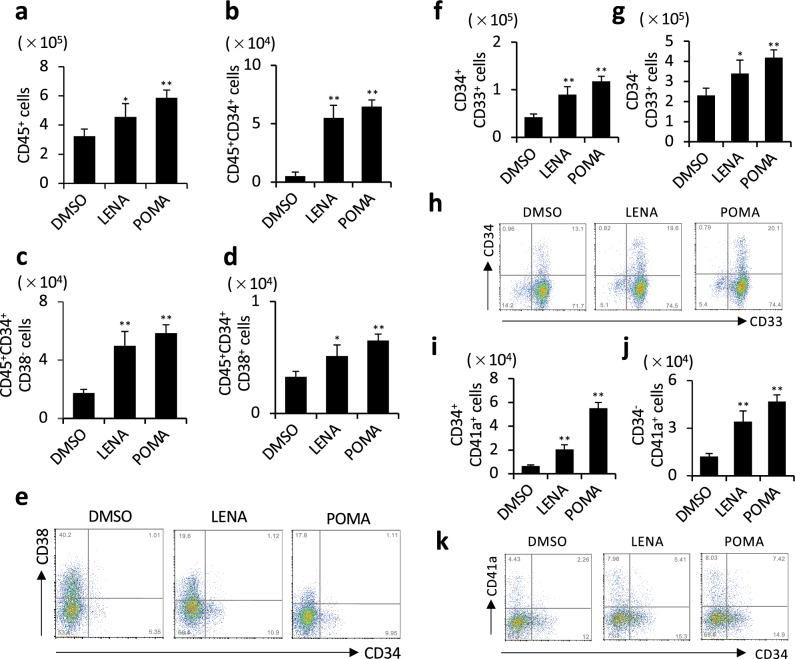
Effects of LENA and POMA on hematopoietic cell populations from CD34^+^ cells in single cultures. **a** Number of overall CD45^+^ cell population with LENA or POMA treatment. **b**–**e** The number of CD45^+^CD34^+^ cell population (**b**), CD45^+^CD34^+^CD38^−^ cell population (**c**), and CD45^+^CD34^+^CD38^+^ cell population (**d**) with LENA or POMA treatment. Representative dot plots of flow cytometric analysis are shown (**e**). **f**–**h** Number of CD34^+^CD33^+^ cell population (**f**) and CD34^−^CD33^+^ cell population (**g**) with LENA or POMA treatment. Representative dot plots of flow cytometric analysis are shown (**h**). **i**–**k** Number of CD34^+^CD41a^+^ cell population (**i**) and CD34^−^CD41a^+^ cell population (**j**) with LENA or POMA treatment. Representative dot plots of flow cytometric analysis are shown (**k**). *n* = 3. Statistical significance is indicated by * (*p* < 0.05) and ** (*p* < 0.01)

In cultures comprised exclusively of CD34^+^ cells maintained in SFEM, the addition of either LENA or POMA significantly increased the overall number of CD45^+^ cells across different lots of CD34^+^ cells (Figs. 3a, S3a). There was also a notable increase in the populations of both CD34^+^CD38^−^ and CD34^+^CD38^+^ cells (Figs. 3b–e, S3b–e). Further analysis indicated a rise in the number of cells within the myeloid lineage (CD34^+^CD33^+^, CD34^−^CD33^+^) (Figs. 3f–h, S3f–h). In addition, there was an enhancement in the population of cells from the megakaryocytic lineage (CD34^+^CD41a^+^, CD34^−^CD41a^+^) (Figs. 3i–k, S3i–k). These findings suggest that LENA and POMA can stimulate the expansion of both early stage and more advanced hematopoietic stem/progenitor cell populations and enhance the generation of myeloid and megakaryocytic cells from CD34^+^ hematopoietic stem/progenitor cells.

### LENA and POMA's modulatory effects on CD34^+^ cell maturation with MSC coculture

**Fig. 4:**
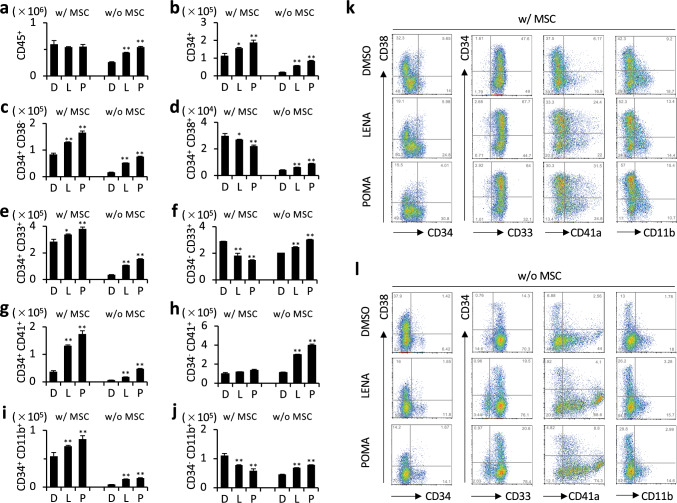
MSC influence on LENA- and POMA-stimulated hematopoietic cell populations derived from CD34^+^ cells in cocultures. **a** Number of overall CD45^+^ cell population with LENA or POMA treatment in the presence (w/ MSC) or absence (w/o MSC) of MSCs. **b**–**e** Number of CD34^+^ cell population (**b**), CD34^+^CD38^−^ cell population (**c**), and CD34^+^CD38^+^ cell population (**d**) with LENA or POMA treatment, in the presence or absence of MSCs. **e**, **f** Number of CD34^+^CD33^+^ cell population (e) and CD34^−^CD33^+^ cell population (**f**) with LENA or POMA treatment, in the presence or absence of MSCs. **g**, **h** Number of CD34^+^CD41a^+^ cell population (g) and CD34^−^CD41a^+^ cell population (**h**) with LENA or POMA treatment, in the presence or absence of MSCs. **i**, **j** The number of CD34^+^CD11b^+^ cell population (**i**) and CD34^−^CD11b^+^ cell population (**j**) with LENA or POMA treatment, in the presence or absence of MSCs. Representative dot plots of flow cytometric analysis are shown (**k**, **l**). *n* = 3. Statistical significance is indicated by * (*p* < 0.05) and ** (*p* < 0.01)

Within CD34^+^ cell cultures in SFEM, the presence of MSCs notably increased the CD45^+^ cell population, underscoring their significant role in enhancing hematopoietic activity (Fig. 4a). However, in these MSC-containing cultures, LENA and POMA did not further influence the increase of CD45^+^ cells (Fig. 4a). The experiments demonstrated that LENA and POMA contributed to an increase in the counts of CD34^+^ cells and their early stage subset, CD34^+^CD38^−^ cells. Conversely, the more differentiated CD34^+^CD38^+^ cells were increased in cultures without MSCs but decreased in cocultures with MSCs (Fig. 4b–d, k, l). This pattern suggests that MSCs play a critical role in maintaining the immature phenotype of hematopoietic stem/progenitor cell populations during treatment with LENA or POMA. Furthermore, increases in CD34^+^CD33^+^ cells were observed with LENA or POMA treatment, while CD34^−^CD33^+^ cell counts decreased in cocultures, indicating that MSCs may moderate myeloid differentiation (Fig. 4e, f, k, l). In MSC cocultures, the presence of either LENA or POMA led to an increase in both CD34^+^CD41a^+^ cells and CD34^−^CD41a^+^ cells. However, while the increase in CD34^+^CD41a^+^ cells was apparent, the rise in CD34^−^CD41a^+^ cell counts was not significant. This suggests that MSCs may influence the LENA or POMA-induced maturation from mature to immature megakaryocytic lineage cell populations (Fig. 4g, h, k, l). For granulocytic differentiation, LENA or POMA increased the counts of CD34^+^CD11b^+^ cells but resulted in a decrease in CD34^−^CD11b^+^ cells in cocultures, suggesting an MSC-mediated suppressive effect on the progression of granulocytic differentiation (Fig. 4i, j, k, l). Overall, in cocultures of CD34^+^ cells with MSCs in SFEM, LENA and POMA enhance the counts of early progenitor cells across various lineages while moderating the maturation of more differentiated cells, reflecting the complex interplay between these agents and the MSC-mediated microenvironment.

### Impact of LENA and POMA on the myeloid maturation of CD34^+^ cells in MSC cocultures

**Fig. 5:**
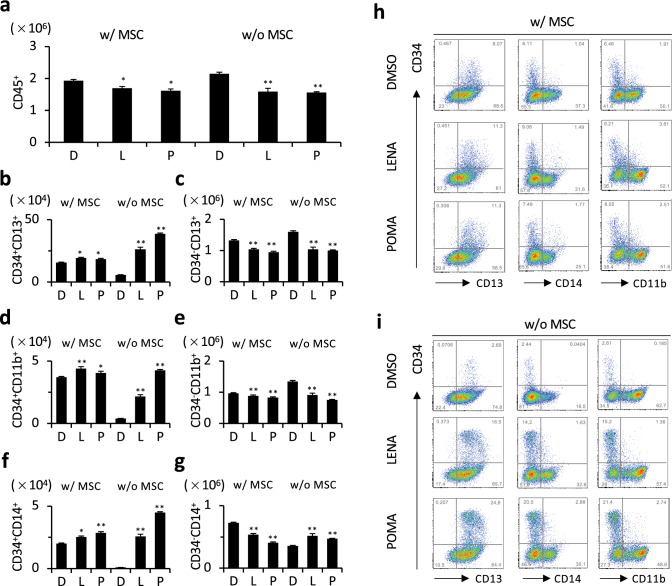
MSC impact on G-CSF-driven myeloid differentiation during LENA- or POMA-induced CD34^+^ cell maturation in cocultures. **a** Number of overall CD45^+^ cell population with LENA or POMA treatment, in the presence (w/ MSC) or absence (w/o MSC) of MSCs. **b**, **c** Number of CD34^+^CD13^+^ cell population (**b**), and CD34^−^CD13^+^ cell population (**c**) with LENA or POMA treatment, in the presence or absence of MSCs. **d**, **e** Number of CD34^+^CD11b^+^ cell population (**d**) and CD34^−^CD11b^+^ cell population (**e**) with LENA or POMA treatment, in the presence or absence of MSCs. **f**, **g** Number of CD34^+^CD14^+^ cell population (**f**) and CD34^−^CD14^+^ cell population (**g**) with LENA or POMA treatment, in the presence or absence of MSCs. Representative dot plots of flow cytometric analysis are shown (**h**, **i**). *n* = 3. Statistical significance is indicated by * (*p* < 0.05) and ** (*p* < 0.01)

Our investigation into CD34^+^ cell cocultures with MSCs, supplemented with G-CSF in SFEM-based complete media, revealed significant impacts of LENA and POMA on the differentiation pathways of myeloid cells. Regardless of the presence of MSCs, the application of LENA or POMA led to a decrease in the number of CD45^+^ cells (Fig. 5a). In addition, there was an observed increase in CD34^+^CD13^+^ cell populations, coupled with a reduction in CD34^−^CD13^+^ cells (Fig. 5b, c, h, i), suggesting that LENA and POMA may disrupt the normal progression of myeloid cell maturation, favoring an earlier progenitor state. This pattern was further demonstrated by an increase in CD34^+^CD11b^+^ cells, while the number of CD34^−^CD11b^+^ cells diminished (Fig. 5d, e, h, i), indicating a targeted inhibition of granulocytic differentiation by these treatments. For cells marking the monocytic lineage (CD14^+^ cells), treatments with LENA or POMA enhanced the populations of CD34^+^CD14^+^ cells and concurrently reduced the number of CD34^−^CD14^+ ^cells within MSC-containing cocultures. Conversely, in the absence of MSCs, an increase in both CD34^+^CD14^+^ and CD34^−^CD14^+^ cell counts was noted (Fig. 5f, g, h, i), emphasizing the complex role MSCs play in modulating the effects of LENA and POMA on monocytic differentiation. In summary, our findings illuminate the inhibitory role of LENA and POMA in the maturation of myeloid cells, specifically affecting the pathways leading to granulocytic (CD11b^+^ cells) and monocytic (CD14^+^ cells) cell differentiation within the context of MSC cocultures.

### Impact of LENA and POMA on erythroid differentiation of CD34^+^ cells in the presence of MSCs

**Fig. 6:**
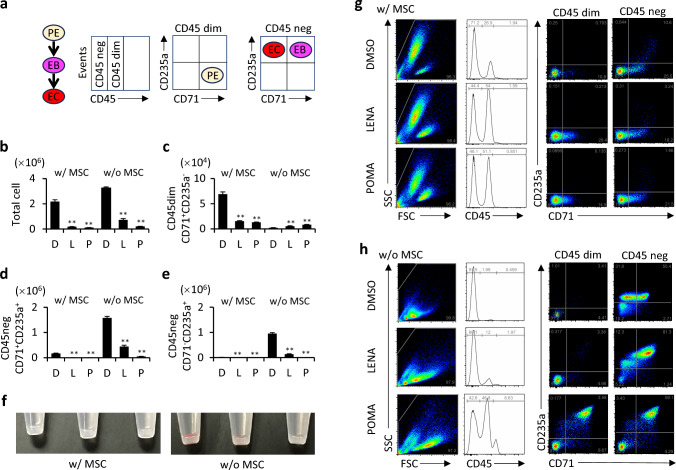
MSC regulation of LENA- and POMA-induced erythroid differentiation in cocultures. **a** Schematic presentation of erythroid differentiation from proerythroblasts (PE, CD45^dim^CD71^+^CD235a^−^) to erythroblasts (EB, CD45^−^CD71^+^CD235a^+^) and then to erythrocytes (EC, CD45^dim^CD71^−^CD235a^+^). **b** Total cell number with LENA or POMA treatment, in the presence (w/ MSC) or absence (w/o MSC) of MSCs. Images of the cell pellet in each culture are shown in panel (**f**). **c**–**e** Number of proerythroblast population (PE, **c**), erythroblast population (EB, **d**), and erythrocyte population (EC, **e**) with LENA or POMA treatment, in the presence or absence of MSCs. Representative dot plots of flow cytometric analysis are shown (**g**, **h**). *n* = 3. Statistical significance is indicated by ** (*p* < 0.01)

Our research has focused on examining the effects of LENA and POMA on erythroid differentiation in CD34^+^ cells. We have documented the phenotypic transitions from proerythroblasts to erythroblasts, and ultimately to mature erythrocytes, as depicted in Fig. 6a. Notably, we observed a significant decrease in the overall cell count in samples treated with either LENA or POMA, independent of the presence of MSCs (Fig. 6b). More specifically, in cultures composed exclusively of CD34^+^ cells, treatment with either LENA or POMA resulted in a reduction in the numbers of erythroblasts (CD45^−^CD71^+^CD235a^+^) and erythrocytes (CD45^−^CD71^−^CD235a^+^), alongside an increase in the number of proerythroblasts (CD45^dim^CD71^+^CD235a^−^), as detailed in Figs. 6b–f, h. Conversely, in co-cultures that included MSCs, treatment with either compound led to a decrease in all three cell types: proerythroblasts, erythroblasts, and erythrocytes (Fig. 6b–f, g). This variation in outcomes between mono-cultures and co-cultures suggests a potential role for MSCs in maintaining more immature cell populations.

## Discussion

Previous pharmacokinetic research has established that the maximum plasma concentration of LENA reaches 1.5 µM in young adults and 2.2 µM in older adults following the administration of a 25 mg dose [20]. Similarly, another study reported that the maximum plasma concentration of POMA is 0.14 µM when a 2 mg dose is administered [21]. The half-life of LENA is approximately 3 h, and it does not accumulate significantly in the body [22]. Similarly, POMA has a half-life of about 7.5 h and also shows no significant accumulation [23]. Given the absence of potential accumulation with consecutive administration of these drugs in clinical settings, we believe that the concentrations used in this study (3 µM for LENA, 0.3 µM for POMA) are appropriate and reflective of clinical conditions. These pharmacokinetic profiles provide a rationale for the concentrations of LENA and POMA used in our study, aligning them with clinically relevant doses for the treatment of hematological disorders. This alignment ensures that our experimental conditions closely mimic the therapeutic context, thus enhancing the relevance and applicability of our findings to clinical settings.

During our examination of hematopoietic cell generation from CD34^+^ cells, we observed that both LENA and POMA significantly increased the population of CD34^+^CD38^−^ cells, while concurrently decreasing the CD34^+^CD38^+^ cell population. These agents also increased the CD34^+^CD33^+^cell population, but reduced the CD34^−^CD33^+^ cell population. These specific effects were noted in the presence of MSCs, highlighting their potential role in retaining early stage cells during LENA- and POMA-induced expansion and differentiation of hematopoietic stem and progenitor cells. Given that CD33 is predominantly expressed on myeloblasts, promyelocytes, myelocytes, some metamyelocytes, and monocytes—and generally absent from band forms and mature granulocytes — the suppression of CD33^+ ^cell differentiation could potentially lead to neutropenia. This is a significant hematological side effect of both LENA and POMA. The potential induction of neutropenia underscores the necessity for careful management of these agents' therapeutic use, particularly in settings where MSCs significantly influence hematopoietic cell dynamics. Previous studies have indicated that MSCs support hematopoietic stem/progenitor cells through both humoral factors, such as CXCL2, and direct cell–cell contact [8–11, 24]. Whether the effect of LENA and POMA involves cell contact or humoral factors, or both, warrants further investigation to elucidate the detailed molecular mechanisms underlying LENA- and POMA-mediated cytopenia. In addition, whether the hematopoietic expansion mediated by these drugs results from an active phase of the cell cycle needs to be elucidated, such as through Ki67 expression level analysis of these cells.

In assays promoting myeloid-differentiation involving MSC co-cultures and the addition of G-CSF, both LENA and POMA were shown to increase populations positive for CD34 and various myeloid markers, including CD11b, while decreasing populations negative for CD34 but positive for various myeloid markers. This points to a retardation of myeloid differentiation towards neutrophils induced by LENA or POMA. This effect persists in cultures without MSCs, except in the case of monocytic differentiation, as indicated by the behavior of the CD14^+^ cell population. The reason for the difference in this specific population may relate to the immunomodulatory capabilities of MSCs [25], which are known to suppress the differentiation and function of monocyte-derived dendritic cells [26].

LENA is widely recognized for its effectiveness in treating anemia associated with MDS characterized by the deletion of the long arm of chromosome 5 [3]. Consistent with prior research [5], our study confirms that both LENA and POMA suppress erythroid differentiation. This suppression was observed regardless of MSC presence; however, the presence of MSCs seemed to enhance the retention of less differentiated erythroid populations. This observation suggests that LENA's clinical benefits in improving anemia in 5q-MDS likely arise primarily from its direct effects on hematopoietic cells. The mechanisms may involve selective cytotoxic actions targeting 5q- clones mediated through the downregulation of critical erythroid transcription factors such as GATA-1 [27], which plays a pivotal role in erythroid development. The additional presence of MSCs in the cellular microenvironment may further modulate these effects, potentially by creating conditions that favor the maintenance of progenitor cells rather than allowing their progression to fully mature erythrocytes.

In our investigation of megakaryocytic differentiation, we observed that treatment with LENA or POMA, in the absence of MSCs, resulted in increased populations of both CD34 ^+^CD41a^+^ and CD34^−^CD41a^+ ^cells. However, when MSCs were present, there was a pronounced enhancement in the CD34^+^CD41a^+^ cell population, while the increase in the CD34^−^CD41a^+^ population was not as evident. This suggests that MSCs may preferentially support the retention of immature megakaryocytic progenitors, specifically CD34^+^CD41a^+^ cells, rather than more mature CD34^−^CD41a^+^ cells. Given that CD41a marks megakaryocytes at all stages of differentiation, our study faced challenges in distinctly categorizing the stages of megakaryocyte development. To address this limitation, future research could utilize colony-forming assays to delve deeper into the formation and differentiation of megakaryocytic colonies [28]. In addition, employing transmission electron microscopy could provide detailed morphological insights into megakaryocytes [28]. These methodologies are expected to enhance our understanding of megakaryocytic development and the responses to treatments with LENA, POMA, and MSCs.

Contrary to the clinical observation that proteasome inhibitors enhance bone formation, which has been substantiated by studies showing direct promotion of osteogenic differentiation of MSCs [29, 30], the effects of LENA and POMA on bone health in patients remain unclear. In our study, both LENA and POMA were shown to reduce the osteogenic differentiation characteristics of MSCs in vitro and in vivo, aligning with findings from a previous in vitro study [31]. However, LENA has been observed to inhibit osteoclast formation by downregulating PU.1 [32], suggesting a potential protective role against bone resorption. This complex interaction suggests that the effects of LENA and POMA on bone remodeling are dependent on the individual's physiological and pathological conditions. The differential impact on osteogenic and osteoclast differentiation pathways indicates that while these agents might diminish bone formation capabilities, they could also confer benefits by reducing bone degradation. This dual role underscores the necessity for a nuanced understanding of their effects within the broader context of bone health and disease management, particularly in conditions involving abnormal bone remodeling.

In summary, MSCs play a critical role in supporting the early stage hematopoietic stem/progenitor cell populations during the treatment of CD34^+^ cells with LENA and POMA. In addition, MSCs aid in the maintenance of various progenitor populations, including myeloid progenitor cells, megakaryocytic progenitor cells, and proerythroblasts. This supportive role of MSCs is particularly significant in the context of cytopenia, a common complication observed in hematologic therapies involving LENA and POMA. Understanding the interactions between MSCs and these therapeutic agents, particularly in myeloma patients [33], could provide important insights into improving treatment strategies and mitigating side effects such as cytopenia.

### Supplementary Information

Below is the link to the electronic supplementary material.

Supplementary file1 (DOCX 17 KB)

Supplementary file2 (PPTX 5848 KB)

## Data Availability

The authors confirm that the data supporting the findings of this study are available within the article and its Supplementary Information at the journal’s website.
