# Supplementary material for: Lenalidomide and pomalidomide modulate hematopoietic cell expansion and differentiation in the presence of MSC
Source: Int J Hematol. 2024 Jul 12;120(3):278–89. doi: 10.1007/s12185-024-03815-y (PMC11362235; doi:10.1007/s12185-024-03815-y)
Supplement: Supplementary file 1 — Supplementary file1 (DOCX 17 KB) [file 12185_2024_3815_MOESM1_ESM.docx]

| **Antibody** | **Vendor** | **Isotype** | **Clone/Product #** | **Application/Fluorochrome** |
| --- | --- | --- | --- | --- |
| CD11b | eBioscience | Mouse IgG1, κ | ICRF44 | FC/PE |
| CD13 | eBioscience | Mouse IgG1, κ | WM-15 | FC/PE |
| CD14 | eBioscience | Mouse IgG1, κ | 61D3 | FC/PE |
| CD34 | BD Pharmingen | Mouse IgG1, κ | 563 | FC/PE |
| CD34 | eBioscience | Mouse IgG1, κ | 4H11 | FC/APC |
| CD45 | BD Pharmingen | Mouse IgG1, κ | 2D1 | FC/FITC |
| CD38 | eBioscience | Mouse IgG1, κ | HIT2 | FC/APC |
| CD41a | eBioscience | Mouse IgG1, κ | HIP8 | FC/PE |
| CD33 | eBioscience | Mouse IgG1, κ | WM-53 | FC/PE |
| CD235a | eBioscience | Mouse IgG2b, κ | HIR2 | FC/PE |
| CD71 | eBioscience | Mouse IgG1, κ | OKT9 | FC/APC |
| Isotype control | BD Pharmingen | Mouse IgG1, κ | MOPC-21 | FC/FITC or PE or APC |
| Isotype control | eBioscience | Mouse IgG1, κ | P3.6.2.8.1 | FC/PE |
| Isotype control | eBioscience | Mouse IgG2b, κ | eBMG2b | FC/PE |

**Supplementary Table 1.** List of antibodies. FC, flow cytometry; IB, immunoblot; PE, phycoerythrin; APC, allophycocyanin; FITC, fluorescein isothiocyanate;

| **Gene** | **Forward primer (5’-3’)** | **Reverse primer (5’-3’)** | **Universal probe (#)** |
| --- | --- | --- | --- |
| Runx2 | ctaccaccccgctgtcttc | aaaaagggcccagttctga | 4 |
| ALP | agaaccccaaaggcttcttc | cttggcttttccttcatggt | 31 |
| GAPDH | agccacatcgctcagacac | gcccaatacgaccaaatcc | 60 |

**Supplementary Table 2.** List of primer sets and universal probes used for real-time PCR. Runx2, runt-related transcription factor 2; ALP, alkaline phosphatase; GAPDH, glyceraldehyde-3-phosphate dehydrogenase.
