# Supplementary material for: Lenalidomide and pomalidomide modulate hematopoietic cell expansion and differentiation in the presence of MSC
Source: Int J Hematol. 2024 Jul 12;120(3):278–89. doi: 10.1007/s12185-024-03815-y (PMC11362235; doi:10.1007/s12185-024-03815-y)
Supplement: Supplementary file 2 — Supplementary file2 (PPTX 5848 KB) [file 12185_2024_3815_MOESM2_ESM.pptx]

## Slide 1
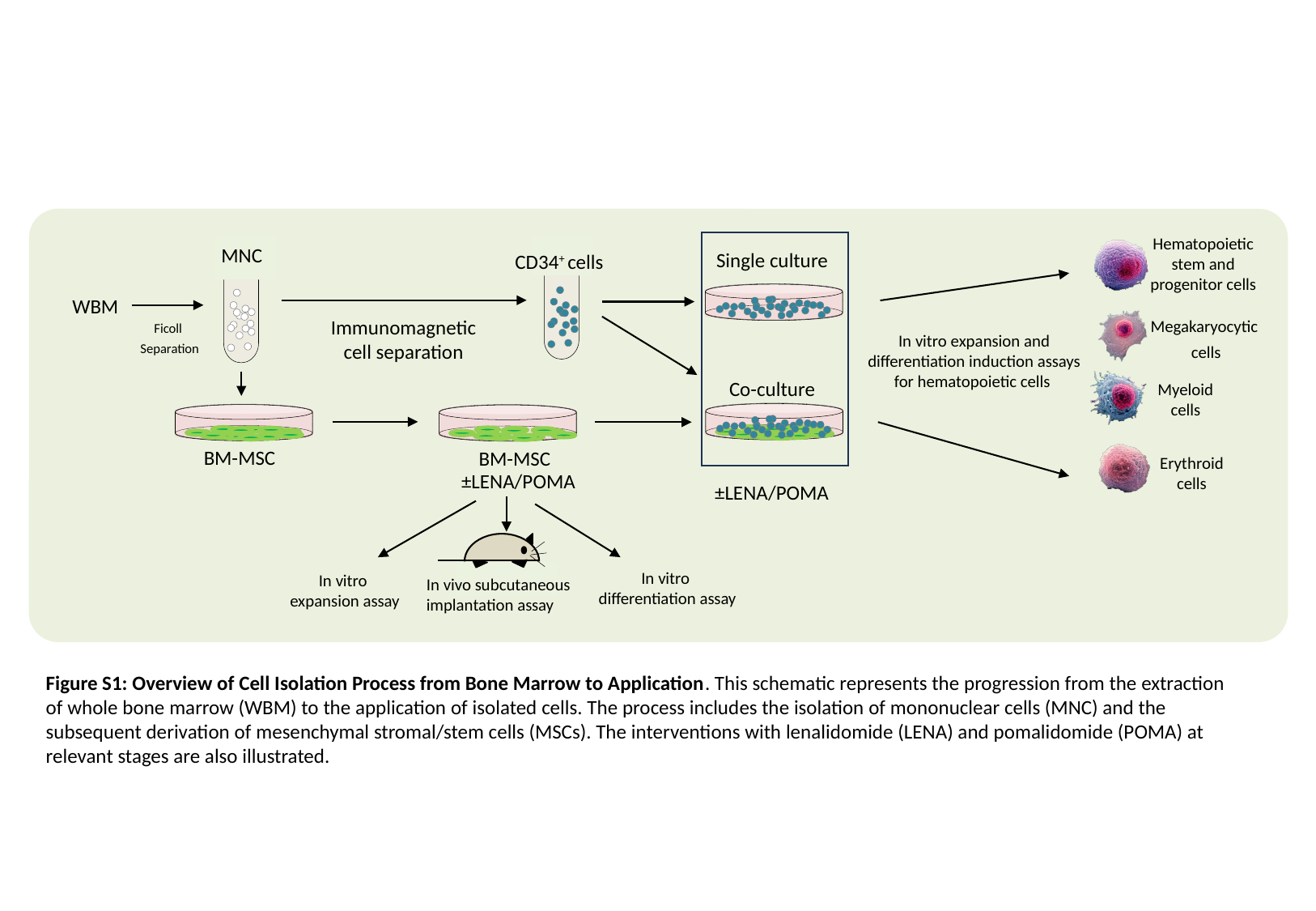

Hematopoietic stem and progenitor cells
MNC
Single culture
CD34+ cells
WBM
Immunomagnetic cell separation
Megakaryocytic
cells
Ficoll
Separation
In vitro expansion and differentiation induction assays for hematopoietic cells
Co-culture
Myeloid cells
BM-MSC
BM-MSC
Erythroid cells
±LENA/POMA
±LENA/POMA
In vitro
differentiation assay
In vitro
expansion assay
In vivo subcutaneous implantation assay
Figure S1: Overview of Cell Isolation Process from Bone Marrow to Application. This schematic represents the progression from the extraction of whole bone marrow (WBM) to the application of isolated cells. The process includes the isolation of mononuclear cells (MNC) and the subsequent derivation of mesenchymal stromal/stem cells (MSCs). The interventions with lenalidomide (LENA) and pomalidomide (POMA) at relevant stages are also illustrated.

## Slide 2
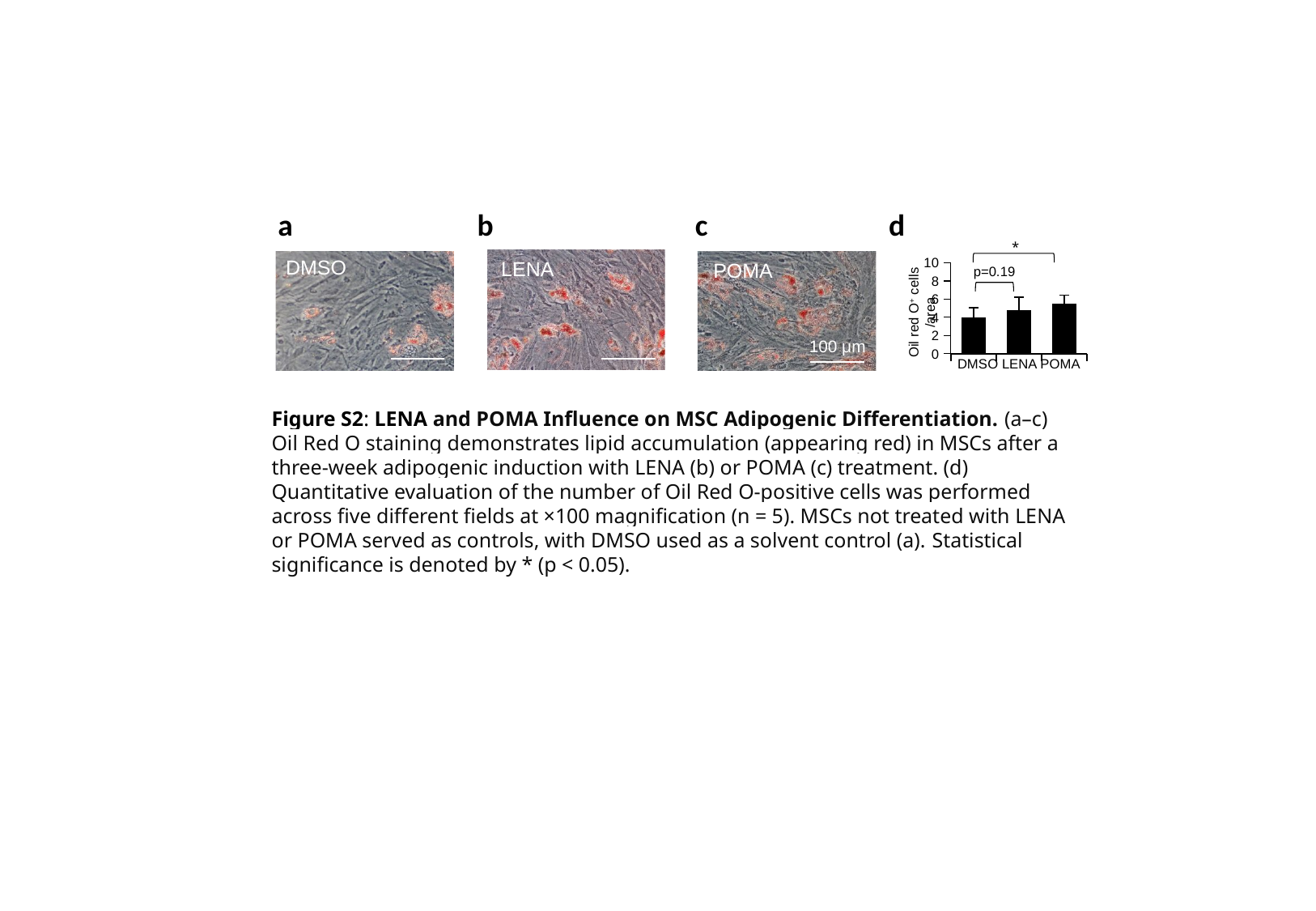

a
b
c
d
*
DMSO
LENA
POMA
### Chart
| Category | |
|---|---|
| DMSO | 4.0 |
| Len3uM | 4.8 |
| Pom1uM | 5.5 |p=0.19
Oil red O+ cells
/area
100 μm
DMSO LENA POMA
Figure S2: LENA and POMA Influence on MSC Adipogenic Differentiation. (a–c) Oil Red O staining demonstrates lipid accumulation (appearing red) in MSCs after a three-week adipogenic induction with LENA (b) or POMA (c) treatment. (d) Quantitative evaluation of the number of Oil Red O-positive cells was performed across five different fields at ×100 magnification (n = 5). MSCs not treated with LENA or POMA served as controls, with DMSO used as a solvent control (a). Statistical significance is denoted by * (p < 0.05).

## Slide 3
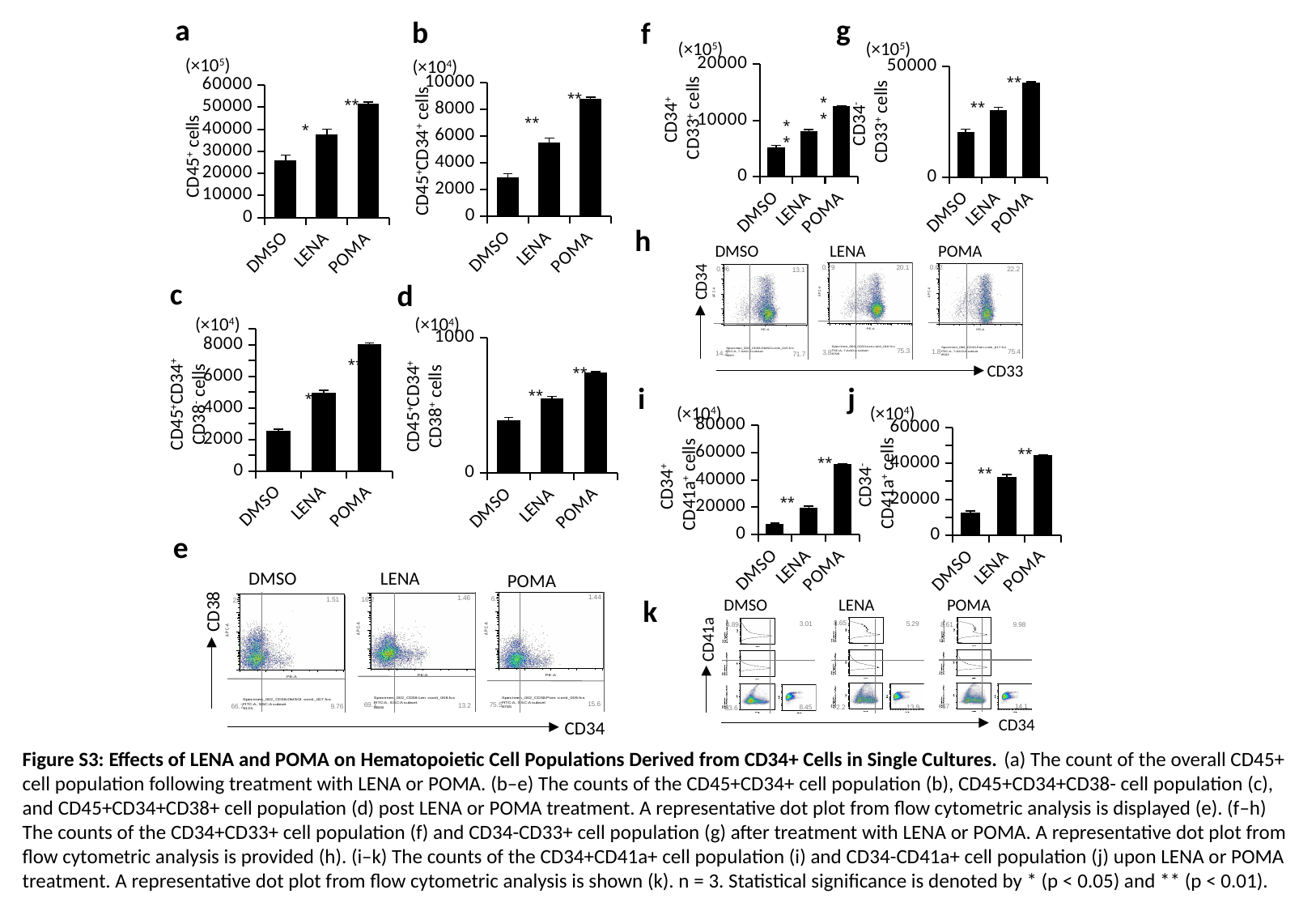

a
g
b
f
(×105)
(×105)
(×105)
(×104)
### Chart
| Category | |
|---|---|
| DMSO | 5284.333333333333 |
| LENA | 8123.75 |
| POMA | 12552.25 |
### Chart
| Category | |
|---|---|
| DMSO | 20556.333333333332 |
| LENA | 30433.75 |
| POMA | 42632.416666666664 |**
### Chart
| Category | CD34+ |
|---|---|
| DMSO | 2924.715266666666 |
| LENA | 5510.3275 |
| POMA | 8796.481099999999 |
### Chart
| Category | CD45+ |
|---|---|
| DMSO | 25951.333333333332 |
| LENA | 37587.5 |
| POMA | 51622.541666666664 |**
**
CD34+
CD33+ cells
**
**
CD34-
CD33+ cells
**
**
*
CD45+CD34+ cells
CD45+ cells
h
DMSO
LENA
POMA
0.62
22.2
75.4
1.8
0.79
20.1
75.3
3.8
0.96
13.1
14.2
71.7
CD34
c
d
(×104)
(×104)
### Chart
| Category | CD45+ |
|---|---|
| DMSO | 391.8651333333333 |
| LENA | 548.7775 |
| POMA | 743.3646 |
### Chart
| Category | CD45+34+38- |
|---|---|
| DMSO | 2532.8501333333334 |
| LENA | 4961.549999999999 |
| POMA | 8053.1165 |**
CD33
**
i
j
CD45+CD34+ CD38- cells
CD45+CD34+ CD38+ cells
**
**
(×104)
(×104)
### Chart
| Category | |
|---|---|
| DMSO | 7811.351333333332 |
| LENA | 19883.787500000002 |
| POMA | 51519.296583333344 |
### Chart
| Category | |
|---|---|
| DMSO | 12690.202 |
| LENA | 32513.1875 |
| POMA | 44447.008375 |**
**
CD34-
CD41a+ cells
CD34+
CD41a+ cells
**
**
e
DMSO
LENA
POMA
k
1.44
6.4
15.6
75.5
DMSO
LENA
POMA
1.46
16.2
69.1
13.2
1.51
22
66.7
9.76
CD38
8.65
5.29
3.01
8.61
4.89
9.98
CD41a
67
14.1
72.2
8.45
13.9
83.6
CD34
CD34
Figure S3: Effects of LENA and POMA on Hematopoietic Cell Populations Derived from CD34+ Cells in Single Cultures. (a) The count of the overall CD45+ cell population following treatment with LENA or POMA. (b–e) The counts of the CD45+CD34+ cell population (b), CD45+CD34+CD38- cell population (c), and CD45+CD34+CD38+ cell population (d) post LENA or POMA treatment. A representative dot plot from flow cytometric analysis is displayed (e). (f–h) The counts of the CD34+CD33+ cell population (f) and CD34-CD33+ cell population (g) after treatment with LENA or POMA. A representative dot plot from flow cytometric analysis is provided (h). (i–k) The counts of the CD34+CD41a+ cell population (i) and CD34-CD41a+ cell population (j) upon LENA or POMA treatment. A representative dot plot from flow cytometric analysis is shown (k). n = 3. Statistical significance is denoted by * (p < 0.05) and ** (p < 0.01).
